# Mechanotransduction: use the force(s)

**DOI:** 10.1186/s12915-015-0150-4

**Published:** 2015-07-04

**Authors:** Ewa K. Paluch, Celeste M. Nelson, Nicolas Biais, Ben Fabry, Jens Moeller, Beth L. Pruitt, Carina Wollnik, Galina Kudryasheva, Florian Rehfeldt, Walter Federle

**Affiliations:** MRC Laboratory for Molecular Cell Biology, University College London, Gower Street, London, WC1E 6BT UK; Chemical & Biological Engineering, Princeton University, 303 Hoyt Laboratory, William Street, Princeton, NJ 08544 USA; Biology Department, Brooklyn College and the Graduate Center of the City University of New York, 2900 Bedford avenue, Brooklyn, NY 11210 USA; Department of Physics, University of Erlangen-Nuremberg, Henkestrasse 91, 91052 Erlangen, Germany; Department of Mechanical Engineering, Microsystems Laboratory, Stanford University, 496 Lomita Mall, Durand Building Rm 102, Stanford, CA 94305 USA; Department of Mechanical Engineering and Molecular and Cellular Physiology, Microsystems Laboratory, Stanford University, by courtesy, 496 Lomita Mall, Durand Building Rm 213, Stanford, CA 94305 USA; Georg-August-University, 3rd Institute of Physics – Biophysics, Friedrich-Hund-Platz 1, 37077 Göttingen, Germany; Department of Zoology, University of Cambridge, Downing Street, Cambridge, CB2 3EJ UK

## Abstract

Mechanotransduction - how cells sense physical forces and translate them into biochemical and biological responses - is a vibrant and rapidly-progressing field, and is important for a broad range of biological phenomena. This forum explores the role of mechanotransduction in a variety of cellular activities and highlights intriguing questions that deserve further attention.

## Four centuries of cells and mechanical forces

### Celeste M Nelson

Cells were first observed under the microscope by Robert Hooke in 1665. That these tiny objects are actually the most fundamental units of life would not be appreciated until the early 1800s, but Hooke’s seminal discovery is striking to us now in the early 21^st^ century for another reason. Amongst physical scientists, Hooke is better known for ‘Hooke’s Law’, the principle of physics that states that the force *F* needed to change the length of an elastic spring by some distance *x* is linearly proportional to that distance (*F = kx*), where *k* is the proportionality constant that describes the stiffness of the spring. Hooke thus made the earliest discoveries that would eventually father two seemingly disparate fields. We now know, of course, that physical forces are fundamental to cell biology.

That cells are subject to the laws of physics - of mechanics - was first postulated by Wilhelm His in the late 1800s [1]. The physical nature of cells and tissues was embraced by embryologists and early cell biologists, as the only tools to interrogate their behavior were mechanical in nature. The discovery of the structure of DNA by Watson and Crick in 1953 ushered in an exciting new era of molecular biology - instead of being considered a physical material, the cell was viewed as a container of genetic material and enzymes. The past 20 years have seen a resurgence of mechanics in cell biology, with new paradigms emerging that have changed our understanding of almost every fundamental cellular process, from cell division to differentiation to morphogenesis.

This new age of enlightenment in mechanobiology has been enabled by technological breakthroughs resulting from collaborations between biologists, physicists, and engineers. We can now estimate the forces that cells exert on their surroundings. Traction force microscopy [2] is one approach to perform this estimation: cells are plated on a compliant substratum (or within a hydrogel [3]) that contains beads that act as fiducial markers. As the cell exerts force on the substratum, the resulting motion of the beads is tracked. The measured bead displacements can then be used to estimate the force exerted by the cells *à la* Hooke’s Law; the actual math involved for a quantitative understanding is more complicated than the equation described above since the physical situation is significantly more complex than the stretching of a spring, but the spirit of Hooke’s equation holds. It is important to note that force is not measured here - it is calculated, and the accuracy of the calculation depends on the resolution of the measurements, the material properties of the substratum, and the validity of the underlying mathematical model.

Other force measurement calculation techniques include micropost arrays and atomic force microscopy (AFM). Micropost arrays actually use Hooke’s Law to calculate the forces exerted by cells on the underlying posts, provided that the deformations of the substratum induced by the cells are small [4]. In AFM, what are really being measured are the mechanical properties of the cell, not the force that the cell exerts. A cantilever probe is used to tap gently on the surface of the cell; the deflection of the cantilever is proportional to the stiffness of the region being tapped. In the mechanobiology literature, these readouts are often mistakenly referred to as ‘tension’.

To detect tension within the cell, advances have been made using molecular sensors. Physically, tension is the pulling force exerted when a one-dimensional chain of objects is pulled apart (the opposite of compression). The recently developed fluorescence-resonance energy transfer (FRET)-based force sensors are intracellular probes that measure tension (not force, per se). These include clever systems that rely on the unfolding of proteins at strategic locations in the cell, including vinculin at focal adhesions [5] and cadherin at adherens junctions [6, 7]. Again, the assumption with these molecular sensors is that the protein behaves as a linear spring, following Hooke’s Law. The validity of this assumption remains to be verified for most cellular contexts.

Almost 400 years after Hooke’s original discoveries, the field is now poised to detail precisely how cells exert physical forces as well as how physical forces alter signaling within cells, a process known as mechanotransduction.

## Forces on cells of all domains of life: mechanotransduction as a common language

### Nicolas Biais

Any assemblage of building blocks - whether animate or inanimate, whether a rock or a human being - needs physical forces to hold itself together. Without the attractive and repulsive forces between atoms, any object we know will just crumble to a nondescript pile of matter. Similarly, without the mechanical interactions between its cells, any multicellular organism would lose its form, functions, and any of the attributes we usually recognize it for. Ever since the seminal work of D’Arcy Thompson [8], there is no denying that physical forces and mechanics are of paramount importance in shaping biological entities, and that importance goes beyond the structural role played by mechanics to hold cells together. The incredible success of molecular biology and the effective explanatory power of reaction–diffusion models have imposed a very chemical mindset to most of our explanations of biological phenomena. Molecular recognition of diffusing cognate molecules (protein-protein or protein-small molecule) is a tenet of biology, but in recent years it has been more and more obvious that the colocalization in time and space of molecules was not always enough to trigger a given biological outcome. In many cases, the existence of forces acting directly on molecules or cells is required in order to trigger the correct biological response. This is in essence what mechanotransduction is: the ability to alter biological outcomes through mechanical forces.

One of the most interesting features of mechanotransduction is that it reveals a new layer of modulation of the interactions between molecules, and a potential global guiding principle for organizing biological entities from molecules to cells. At the same time, as new technological advances have enabled us to measure and apply forces on cells and molecules (optical tweezers, magnetic tweezers, and lithography to name a few examples), we have come to realize how pervasive the role of physical forces is. Mechanotransduction, defined as the modulation of biological fates by physical forces, has been found to occur in all corners of the biological realm and with an extremely rich and diverse set of mechanisms. Some of these mechanisms are very similar across all domains of life, as in the case of the mechanosensitive channels that allow physical stimuli on or across membranes to control the flow of molecules across these membranes: flow that can in turn release osmotic pressure or trigger another signaling pathway [9–11]. Some are more specific to a given subset of cells. As an example, the role of the mammalian cell cytoskeleton in responding to physical cues such as the rigidity of its environment is one of the most studied examples of mechanotransduction.

Thanks to their cytoskeleton, mammalian cells can easily exert forces in the nanoNewton range on their surroundings and sense the mechanics of cells or substrates around them [12]. For mammalian cells, physical forces play a direct role in important biological choices such as stem cell differentiation, motility or tumor formation [13–15]. Only some of the mechanisms of this complex system have been elucidated. Some exemplify direct coupling between chemical signaling and mechanical forces: stretching of some molecules of the focal adhesion exposes either cryptic binding sites or cryptic phosphorylation sites, thus triggering signaling pathways [16, 17]. Others represent responses to physical forces that allow for adaptation of a cell and its cytoskeletal network to external changes of stiffness in less than 100 ms [18]. The physical tension of the plasma membrane can also play a role as an orchestrator of many cellular events [19]. Note that in all instances, the origin of the forces is not important, just that these forces are present. For instance, in the case of the development of the *Drosophila* embryo, forces resulting from internal motions of cells control cellular fate and expression of developmental genes. By altering these forces, one can alter cellular differentiation [20].

Going back to the case of the response of mammalian cells’ cytoskeletons, the recruitment of actin seen at focal adhesion points can also be at least partially recapitulated by artificially exerting forces on other locations of the cells [21]. If it now seems obvious that mechanical cues from mammalian cells’ surroundings and between these cells are crucial for short- and long-term normal biological behavior, the potential mechanical impact of the cells of many bacterial species is largely overlooked. We have known for a long time that we humans are outweighed 10 to 1 in numbers of cells by the microbiome that we carry with us [22]. We now know that we are also outweighed 25 thousand to 5 million in terms of genes [23]. And the data about the modes of interactions of all these bacteria cells with our own cells is still quite scarce. So could it be that many bacteria are using mechanotransduction to interact with our cells? *Neisseria gonorrhoeae*, the causative agent of gonorrhea, has emerged as a paradigm of mechano-micro-biology: the study of the role of physical forces in microbes. The long retractable polymers that emanate from the bodies of these bacteria, named type IV pili, enable them to exert physical forces reaching the nanoNewton range on their surroundings, the same amplitude of forces that mammalian cells exert on their own surroundings [24]. Similarly to the recruitment of actin and other molecules at focal adhesion points, the forces exerted by *N. gonorrhoeae* cells trigger accumulation of actin and other proteins, events critical to colonization of the host [25, 26]. Thus, *Neisseria* and human cells appear to be engaged in a physical cross-talk where bacterial cells have at least partially co-opted mechanotransduction pathways from human cells.

Despite our often detailed understanding of the biochemical reactions that control cellular fates, or maybe because of it, we may overlook the fact that mechanical forces are a powerful means to modulate or override many of these biochemical reactions. Whether members of the eukarya, bacteria or archaea domain of life, all cells share a genetic material made of DNA, but also need to interact physically with their surroundings. Through evolution, many different types of cells have developed mechanisms that can intertwine mechanical forces and biochemical reactions. Not only are those mechanisms essential for the survival of the cells, but they also provide an incredible platform for interaction between cells of all domains. As soon as a cell possesses a way to exert mechanical forces, it will have the ability to modulate the functions of other cells (as we have seen in the case of *Neisseria*). There are many examples of cells from one domain (for instance bacteria) which have evolved toxins or effectors with the ability to hijack complex molecular machinery from cells from another domain (for instance eukarya), but these modes of interactions rely on specific molecular interactions and require a long evolution to be put in place. On the other hand, mechanical forces constitute a natural common language between cells of all domains that can easily be modified. Studying the mechanical interactions between cells of different domains that have co-evolved for a long time, as, for instance, human cells and the members of the human microbiota, will help us to delineate the full modalities of mechanotransduction.

## Force transmission via non-specific friction

### Ewa K Paluch

Specific attachments of cells to their substrate, mediated by dedicated proteins such as integrins or cadherins, have long been considered paramount for cell migration. Yet, recent studies demonstrate that effective cell movement can occur in the absence of such specific attachment points. In a seminal paper in 2008, Lammermann *et al*. [27] showed that dendritic cells can migrate in the lymph node or in collagen matrices in the complete absence of integrins, demonstrating that, in three-dimensional environments, low non-specific adhesion can be sufficient to drive migration. More recently, two experimental studies identified myosin activity and confinement as key parameters favoring a switch to low-adhesion modes of migration in cultured cells and *in vivo* [28, 29].

Several theoretical studies have explored possible mechanical bases of force transmission during migration without specific adhesions. An important requirement for this type of movement is three-dimensional confinement. Indeed, without confinement, thermal fluctuations would prevent sustained contact between the cell and the substrate, which is required for force transmission, and the cell would ‘drift away’, as no specific adhesions are there to anchor it to the substrate [30]. Various theoretical mechanisms of force generation and transmission have been proposed, including chimneying, where cells push themselves off the substrate like an alpinist climbing a rock cleft [31]; intercalation, where lateral protrusions insert into gaps in a discontinuous three-dimensional matrix, thus providing anchors for traction force generation [32]; and non-specific substrate friction that could mediate intracellular forces [33]. In a recent study, we combined theory and experiments to investigate the origin and magnitude of the forces involved in the migration of Walker carcinosarcoma cells, which do not rely on specific adhesions and display active migration in confinement [34]. We could show that Walker cells move using non-specific friction that transmits to the substrate forces generated by contractile acto-myosin flows at the cell cortex. Interestingly, we found that the forces involved are orders of magnitude lower than during specific-adhesion-based migration [34]. Even in conditions of high substrate friction, Walker cells exert stresses lower than 1 Pa, and rapid cell movement is still observed with stresses of a few mPa, strikingly less than the 0.1-5 kPa stresses typically exerted at integrin-mediated adhesions (see also the piece by Ben Fabry in this forum).

As the conditions under which cells display adhesion-independent migration are progressively being unveiled, many important questions arise. For instance, it remains unclear whether any migratory cell can migrate without specific adhesions or if friction-based migration is restricted to certain cell types. From a mechanical standpoint, the finding that the forces driving friction-based migration are orders of magnitude lower than the forces involved in integrin-mediated migration raises the puzzling question of the biological function of the strong forces exerted at integrin-mediated adhesions. One can speculate that these forces primarily function to sense substrate stiffness, which is the basis of durotaxis and can guide differentiation of stem cells (see also the pieces by Ben Fabry and Carina Wollnik *et al*. in this forum). Furthermore, strong attachment forces may be required for cells migrating against a flow, such as in blood vessels. Another key question is the molecular basis of friction. It is unclear whether it depends on the chemistry of the cell surface and the substrate only, or if geometric features such as substrate rugosity and geometry might also play a role. Finally, it will be exciting to elucidate in what physiological contexts friction-based migration occurs *in vivo*.

## Acto-myosin cycling kinetics and focal adhesion reinforcement drives cellular durotaxis

### Ben Fabry

Durotaxis describes the movement of cells along a stiffness gradient of the substrate. Similar to chemotaxis where cells migrate towards a concentration gradient of chemokines, durotaxis is thought to be important for tissue formation during embryogenesis, or the migration of cells during wound healing, inflammation, and metastasis. Obviously, durotaxis cannot be fully understood without a basic understanding of cell migration. Migration, in turn, cannot be understood without some understanding of the underlying fundamental processes: cell adhesion (and de-adhesion), spreading, and contraction. The canonical picture of how cells crawl on a planar substrate is that of a sequential four-step process [35]. First, cells form protrusions at the leading edge, driven by actin polymerization. Second, these protrusions are attached to the substrate through the formation of focal adhesions. Third, these focal adhesions are connected to actin stress fibers that are tensed through the contractile activity of myosin motors. Finally, the focal adhesions at the rear end of the cells de-adhere under the influence of time and contractile force. Each of these processes can contribute to durotaxis.

Focal adhesions generate friction between the cell and the substrate. Stronger and longer-lasting adhesions give rise to higher friction and thus result in a lower speed of cell migration. As discussed in more detail below, cells form stronger adhesions and consequently migrate more slowly on stiffer substrates [36]. If we consider that cell migration on a mechanically isotropic substrate is a directionally random process, it follows that cells spend less time in regions with low stiffness, and thus more time in regions with higher stiffness. The net effect is durotaxis, and to understand this, we need to understand why adhesions become stronger on stiffer substrates.

For reasons that are still not fully known, adhesions are reinforced (they become larger and stronger) under mechanical load [37, 38]. The mechanical load equals the internal contractile stress of the cytoskeleton and at the same time the external substrate tractions. Cells usually generate higher tractions on stiffer substrates [39]; hence, adhesions also become stronger on stiffer substrates [36]. Thus, we next need to understand why tractions, and cell contractility, are stiffness-dependent.

As cells contract, the resulting traction forces deform the underlying substrate. The softer the substrate is, the more it deforms. Large deformations, however, pose a problem for the cell for two reasons, both of which were first discovered in muscle tissue. First, the force-generating contractile apparatus of the cells has to shorten, which reduces the force it can generate because the overlap between actin and myosin filaments becomes suboptimal [40]. Second, larger deformations require a larger speed of contraction, and more and more of the myosin-generated forces are wasted to overcome the internal friction with actin [41]. Thus, cells cannot keep up large traction forces on soft substrates, with the consequence that focal adhesions become instable, which allows the cell to migrate faster until it reaches a region of higher substrate stiffness.

This physical picture of durotaxis as presented here is of course highly simplified and neglects important biological details. For example, myosin activation is not constant in a cell but is actively controlled by force-dependent signaling cascades that originate at focal adhesions [42]. Nonetheless, key aspects of durotaxis can be explained without such complex biochemical signaling events. All that is needed are two force-sensitive processes. One appears to be the dependence of myosin-generated cytoskeletal forces on the sliding speed, which is governed by acto-myosin crossbridge cycling kinetics [41], and the other appears to be the stress-dependent reinforcement of focal adhesions, which may also be governed by a simple physical mechanism, namely the catch-bond kinetics reported for focal adhesion proteins [43]. Given the fundamental importance of durotaxis for essential cell behavior in the living organism, it may be sensible that it relies not on complex signaling cascades that can be easily deregulated, but on robust physical principles.

## May the force (deformation) be with you

### Jens Moeller and Beth L Pruitt

#### Do cells sense and respond to forces or deformations?

**Fig. 1.**
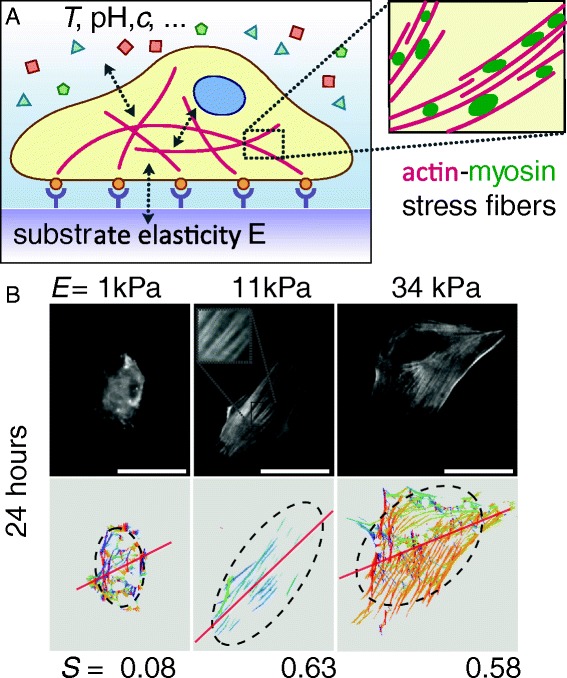
Hooke's law for linear elastic engineering materials compared to complex material models for biological specimens. The ratio between applied stress σ (force/area) and resulting strain ε (deformation) is described by the elastic modulus E for homogenous, isotropic, linear elastic materials. For biological specimens, the material model assumptions are more difficult and depend on the specific system. Proteins, cells and tissues consist of multiple heterogeneous, anisotropic building blocks of various length scales that are hierarchically organized and exhibit rate-dependent, non-linear, viscoelastic stress–strain responses. Comparison of mechanical properties across systems and among different testing methods requires careful assessment of testing conditions and calibration schemes, which are not yet standardized

Cells within tissues are subjected to exogenous, physiological forces, including fluid shear stress or mechanical load, while at the same time cells exert acto-myosin-generated contractile forces to the extracellular matrix (ECM) and to neighboring cells via cell-ECM and cell-cell adhesions [44]. Hooke’s Law and Newton’s Laws of equilibrium readily relate the linear extension of a ‘spring’ to forces, and using appropriate material models we can further relate forces to stresses (force/area). All mechanical measurements revolve around exquisitely precise displacement measurements, yet these displacement data must be converted to estimate force via a set of material deformation models [45]. By necessity, these models are oversimplified because proteins, cells, and tissues present highly anisotropic, heterogeneous, nonlinear mechanical properties that vary widely and depend on the composition, architecture, and environmental conditions, as well as the direction, nature and rate of load application [46]. But do not despair, for while ‘essentially, all models are wrong, some are useful’ [47]. Although we need a material model to estimate forces and stresses, we can directly observe substrate displacements and calculate strains (changes in length/original length) or three-dimensional deformation fields (Fig. 1). To unravel how cells convert these mechanical cues into biochemical signals (mechanotransduction), we must also consider how the structures of proteins and protein networks are altered upon mechanical load. The cellular microenvironment consists of protein networks of varying biochemical and physical properties, including matrix composition, dimensionality and stiffness, all of which have been shown to co-regulate cell function, differentiation, tissue homeostasis and organ development [48, 49].

#### ECM remodeling provides a force-feedback loop

Not only do cell mechanical properties exhibit rate-dependent behaviors such as non-linear viscoelasticity or thermodynamic instabilities, but the substrate itself also changes with time. Cells secrete and remodel the ECM that comprises their tissue microenvironment in a load history-dependent manner. For example, bone resorption by cell-secreted proteases as in microgravity results in more porous, weaker ECM networks while bone growth by cell-secreted and remodeled ECM reinforcement occurs with weight-bearing exercise [50]. Variations in ECM biophysical properties within or across a tissue are not only graded in their composition, crosslinking, dimensionality and stiffness by the cells that created them, but these properties also feedback on cell responses via mechanically mediated cell signaling pathways and enable long-range signaling by cells through the ECM. The biochemical and physical properties of cell-generated ECM protein networks (for example, collagens and laminins in basement membranes or collagens, fibronectin and elastins in blood vessels) co-regulate cell functions such as motility, proliferation, apoptosis [51], stem cell differentiation [13] and organ development [48]. Different cell types show preferences for substrates of different rigidity, which in turn can elicit different cell-ECM traction forces [52]. Unlike inert materials with fixed properties, living cells remodel themselves and their environment under chronic loading and also actively generate mechanical forces through actomyosin-generated tension in the cytoskeleton.

#### How do molecular mechanisms integrate to cellular mechanotransduction?

Even though we can relate the deformations of the cells and the cellular microenvironment to forces, mechanically mediated signaling at the molecular level is also fundamentally governed by deformation and rate sensitivity. Binding kinetics, protein crosslinking and availability of binding sites depend on both the protein sequence and protein conformation, a function of thermodynamic energy. Changing protein conformations can encode distinct functional states with different ligand binding affinities or binding availability by exposing cryptic binding sites for other binding partners. For example, the rod domain of talin, a focal adhesion protein linking integrins to the actin cytoskeleton, undergoes large conformational changes under acto-myosin-generated forces and exposes cryptic binding sites for vinculin [53]. Vinculin recruitment in turn contributes to a reinforcement of the focal adhesion complex to transduce higher load between the cells and the ECM [54]. Similarly, actomyosin-generated tension facilitates vinculin binding to a cryptic binding site in α-catenin in cell-cell adherens junctions to regulate tissue organization [55]. Within the fibrous ECM, fibronectin unraveling is controlled by Rho-mediated cell contractility to expose cryptic self-assembly sites and binding sites for other proteins and growth factors [56, 57]. All those mechanosensitive proteins consist of multiple domains with a range of threshold unfolding loads. At appropriate force thresholds, stiff protein domains (β-sheets, α-helices, barrels) first reorient in the direction of loading as flexible linker chains connecting them (turns, loops, hairpins) stretch and rotate; the individual domains unfold in the order of their stiffness and thus contribute to highly nonlinear force-displacement behavior as proteins can unfold up to >10 times their equilibrium length. Most of the protein force spectroscopy studies quantified force thresholds for these phenomena through a complex set of assumptions both about the measurement tools and the sample to arrive at forces at the scale of picoNewtons in such load-bearing proteins as cadherin [6] and vinculin [5]. While pN molecular forces may be sufficient for mechanically switching individual protein functions or binding affinities, aggregated forces measured at the cellular level are much higher and result in deformation on the surrounding ECM on the order of several cell lengths. Indeed, fibrous scaffolds transmit tension over long distances through their rope-like interconnections [58].

#### How can we measure deformations (forces)?

Applying or measuring displacements (and inferring forces from these measurements) across the length scales of proteins, cells, and tissues requires a range of techniques and several biochemical sensors and microfabricated devices have been developed for this purpose. Optical and magnetic tweezers, Förster Resonance Energy Transfer (FRET) molecular tensions sensors, and atomic force microscopy (AFM) are widely used to study conformational changes of individual mechanosensitive proteins under mechanical load [59], while optical stretchers, micropipette aspiration, AFM and microelectromechanical systems (MEMS) enable single cell mechanobiological studies (see [60] for a review). Meanwhile, traction force microscopy of fiducial markers embedded in compliant substrates and microfabricated post arrays are commonly used to measure the displacement fields of single cells and microtissues [61]. Given the dynamic nature of the state of cells, ECM and proteins, estimates of small forces or heterogeneous mechanical properties are not easily compared between labs using the same method, let alone across methods, and standardized calibration and measurement schemes are needed. Nevertheless, the variability of life is perhaps greater still, and thus mechanobiologists can learn a great deal from appropriately designed experiments and controls to look for relative changes in a consistent framework of measurements and models.

## Mechano-guided differentiation of human mesenchymal stem cells: actomyosin stress fibers as collective mechanosensors

### Carina Wollnik, Galina Kudryasheva and Florian Rehfeldt

It is nowadays well acknowledged that mechanical stimuli can be as important for cells as traditional biochemical cues [62]. They also impact the efficacy of drugs [63] and can influence the morphology and growth phase of cellular aggregates [64]. Especially striking are the experiments by Engler *et al*., demonstrating that substrate elasticity can direct differentiation of human mesenchymal stem cells (hMSCs) towards various linages (neural, muscle, bone) [13]. Here, it is particularly interesting that mimicking Young’s modulus of the *in vivo* environment drives naïve adult stem cells towards the respective cell type. While the initial cue (elastic properties of the matrix) and the overall outcome (changes in transcription) are well-defined, the integration of the mechanical stimuli into biochemical signaling pathways is still not fully understood. Recently, there is mounting evidence that direct mechanical coupling to and perturbation of the nuclear envelope and the nucleus might be an alternative or additional route to alter gene regulation [65].

**Fig. 2.**
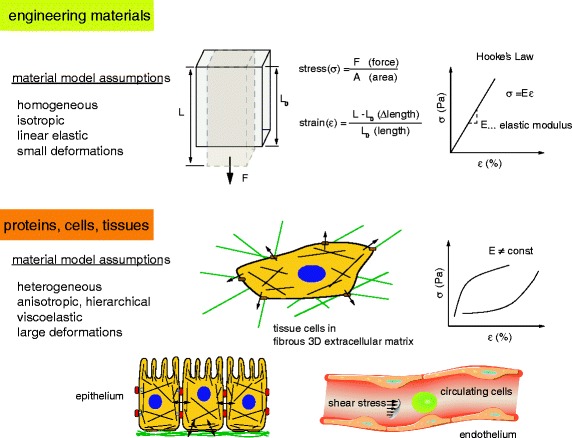
Acto-myosin stress fibers are key mechanical regulators in cell-matrix mechanosensing.** a** Sketch of a cell adhering to a substrate of elasticity *E*. Actomyosin stress fibers (magnified in the inset zoom) are connected via focal adhesions and extracellular matrix proteins to the micro-environment and generate contractile forces that enable the cell to sense the mechanical properties of the substrate. The cytoskeleton is also connected to the nuclear lamina, thus providing a direct mechanical route to gene regulation. Adapted from [67] with permission from The Royal Society of Chemistry. **b** Non-monotonic dependence of stress fiber structure quantified by an order parameter *S* of hMSCs grown on substrates of different elasticity *E* can be used as early morphological marker for mechano-guided differentiation. Scale bar is 50 μm. Adapted from [70] with permission from the Nature Publishing Group

To explain such a mechanical pathway, it is essential to understand how forces can be transmitted to the nucleus. Actomyosin stress fibers are key players in cell adhesion and cell-matrix interactions as they anchor at focal adhesion sites and create cellular contractility (Fig. 2a) [66, 67]. Because they also connect directly to the nuclear lamina [68, 69], stress fibers are able to pass on stress and strain and deform the nucleus [65]. A closer look at the actomyosin filaments in hMSCs revealed that quantification of their structure and organization by means of an order parameter *S* showed significant differences with respect to the substrate elasticity at an early stage of mechano-induced differentiation (24 hours) [70, 71]. On soft (1 kPa) and rigid (34 kPa) substrates the stress fibers are organized more isotropically (*S* = 0.08 and 0.58, respectively), while on 11 kPa substrates (an intermediate elasticity and matching the *in vivo* stiffness of relaxed muscle) the actomyosin bundles were parallel aligned and showed high anisotropy as indicated by an order parameter *S* = 0.63 (Fig. 2b). This early morphological marker can be understood in terms of a collective mechanosensor and is experimentally observable long before lineage-specific genes are upregulated, a process that usually takes several days [72]. Another recent study used this quantitative order parameter analysis to determine the effect of substrate elasticity on differentiating myoblasts [73], indicating that mechanical stimuli and respective change of cytoskeleton structure do play a role in differentiation of more than one cell type.

Such significant differences in macroscopic or global structures are most likely induced by distinct molecular compositions of the actomyosin stress fibers on the nano-scale, a hypothesis that might be confirmed in the near future by super-resolution microscopy methods now available. It is important to note that this type of analysis is now done with fixed cells at distinct times, just like one would do for protein or transcription analysis. To get a better insight into the complex and also highly dynamic processes it will be of paramount importance to follow the temporal evolution of stress-fiber structure and organization followed by live cell imaging and quantitatively extract parameters of the kinetics.

Analyzing transcription profiles and biochemical signaling cascades is essential to finally elucidate the complex processes underlying mechanosensory phenomena. However, the micro- and nano-structural properties of those mechanically active structures contain valuable information on the route from outside mechanical signals to inside biochemical regulation. In the same way that cell biology relied for a long time on descriptive phenotyping by analyzing global cell shape, we should use the additional information of the morphology of stress fibers to complete our picture of mechano-guided differentiation of hMSCs.

## Reversible adhesion in climbing animals - is it similar to cell adhesion?

### Walter Federle

Not only cells but also many climbing animals such as insects, spiders and geckos are able to move around in their environment, yet have to resist detachment forces by forming adhesive contacts. These animals possess special attachment structures on their feet that allow them to cling to substrates. When climbing on trees they may be challenged by the forces of gravity and wind, or by predators trying to dislodge them [74]. Cells, in turn, can be exposed to shear flows in blood vessels or to tensile stresses within tissues. Many animals can climb in a three-dimensional environment such as the forest canopy by repeatedly attaching and detaching their feet, whereas cells can migrate on substrates and within tissues by forming new adhesive contacts at their front and releasing them again at their trailing edge [75]. By way of these functional similarities, it is perhaps natural to compare cell adhesion with the adhesion of climbing animals. Is cell adhesion similar to the adhesion of insects, spiders and geckos?

#### Comparison of adhesive strength

Starting with the *physical mechanism* of adhesion, both cells and climbing animals take advantage of van der Waals forces [76, 77]. These forces only become significant when two objects are in intimate contact with separation distances less than approximately 10 nm [78]. The contacts formed by cell adhesion molecules such as integrin or cadherin and those of gecko adhesive hairs are likely within this range [79, 80]. In addition to van der Waals forces, cell adhesion is strongly dependent on electrostatic forces comprising hydrogen bonds, double layer forces and forces between charged domains of interacting proteins [79]. An important role of electrostatic forces via contact electrification has also been proposed recently for the adhesion of geckos [81], and similar mechanisms are possible for insects [82]. Although more evidence is needed to confirm the extent to which contact electrification contributes to animal adhesion, both cells and climbing animals are probably affected by electrostatic interactions. In addition, the adhesion of many climbing animals involves capillary forces, arising from tiny amounts of fluid secreted into the adhesive contact zone [83–87]. As cells live in a watery environment, this adhesive mechanism is usually absent in cells.

**Fig. 3.**
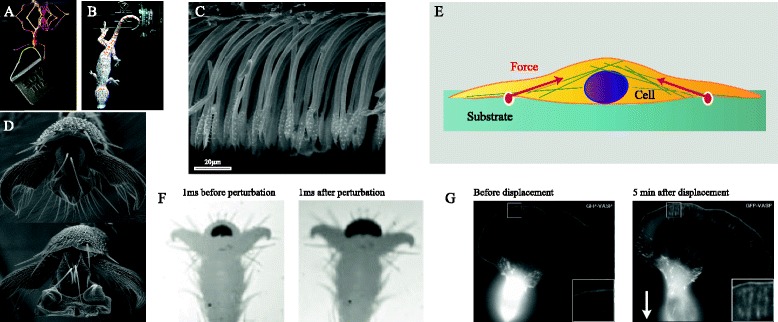
Surface adhesion in climbing animals and cells.** a** Weaver ant (*Oecophylla smaragdina*) carrying more than 100 times its body weight upside-down on a smooth surface (photo: Thomas Endlein). **b** Tokay gecko (*Gekko gecko*) attached by a single toe to a tilted glass surface. Reproduced from [130] with permission from the *Journal of Experimental Biology*. **c** Lateral view of adhesive setae in a longhorn beetle (*Clytus arietis*) showing non-adhesive orientation of seta tips and anti-adhesive corrugations on the dorsal side. Reproduced from [131] with permission from the *Journal of Experimental Biology*. **d** Weaver ant adhesive pad in the retracted (top) and the extended position (bottom). Reproduced from [114]. **e** Adherent cell on a deformable substrate. Inward forces are transmitted via the cytoskeleton and the focal adhesions to the substrate. Adapted from [75]. **f** Rapid increase in adhesive contact area in stick insects (*Carausius morosus*) in response to a rapid displacement of the substrate. Adapted from [121]. **g** B16 melanoma cell (expressing fluorescent marker for focal adhesions) before and 5 minutes after displacement of cell body by a microneedle (direction shown by arrow), showing growth of peripheral focal contacts in the region opposite the cell body (enlarged in insets), stimulated by tension. Reproduced from [123] with permission from the *Journal of Cell Science*

Given that both cells and whole animals adhere by van der Waals forces and probably electrostatic forces, one might expect their adhesive stresses to be of similar magnitude. Let us briefly consider the consequences if this were indeed the case: depending on the geometry, adhesion forces scale with the length or the area of adhesive contacts [88]. For example, while the pull-off force for a suction cup is proportional to its area, the force needed to peel off a piece of Scotch tape depends on its width. For isometric organisms, weight increases with the cube of linear dimensions, and therefore faster than area- or length-specific adhesion. As a consequence, adhesion per body weight is expected to decrease for larger animals. Despite their relatively large body size, however, geckos can easily hang from a single toe, and weaver ants can carry more than 100 times their own body weight whilst walking upside down on a smooth surface (Fig. 3a, b). Clearly, these animals use only a small fraction of their body surface area (that is, the adhesive organs on their feet) for attachment, and at least the gecko does not seem to employ any specific adhesion molecules to achieve high levels of forces.

Cells have an average mass of approximately 1 ng [89] and are thus 5 to 11 orders of magnitude lighter than geckos and insects. Their weight-specific adhesion should thus be enormous in comparison to animals. Moreover, about half of a cell’s surface is in relatively close contact with the substrate, a much larger proportion than for a climbing animal. Thus, one might expect cells to adhere very firmly to any substrate and to one another, even without any specific adhesion molecules, potentially limiting or preventing locomotion. Are the adhesive stresses of cells and climbing animals indeed comparable, and how do they compare with typical levels for van der Waals forces?

van der Waals forces are considered weak intermolecular forces, but they still can produce maximum contact strengths of the order of 100 MPa, sufficient for a 1 cm^2^ contact to support the weight of a small family car [76]. Table 1 shows that the stresses measured for animal adhesives are at least two orders of magnitude below these theoretical strength levels, probably a result of stress concentrations and surface contamination. However, adhesive and shear stresses of cells are much smaller still, by several orders of magnitude. Why are stresses so much smaller for cells, although they use similar molecular forces for attachment?

**Table 1:** Shear and adhesive strength of animal adhesive pads in comparison with single cells

	Strength (kPa)	Source
Shear forces		
Gecko seta: real contact area	53,300	[124, 125]
Gecko seta: projected contact area	2,880	
Beetle pad: real contact area	681	[112]
Beetle pad: projected contact area	259	
Weaver ants	405	[126]
Stick insects	299	[112]
Barnacles	10-300	[127]
Fibroblast cells (whole)	0.048	[128]
Fibroblast cells (focal contacts)	5.5	[103]
Adhesion		
Gecko seta: real contact area	10,700	[124, 125]
Gecko seta: projected contact area	576	
Beetle pad: real contact area	86.9	[112]
Beetle pad: projected contact area	35.5	
Stick insect pad	44.6	[112]
Barnacles	100-1000	[90]
Ants	~50	[126]
Endothelial cells	0.56-1.1	[129]

Firstly, cells live in a watery environment. Water not only provides viscous ‘squeeze-out’ resistance that makes it harder for objects to come into close contact, but it also shields surface charges, and intervening layers of water reduce van der Waals forces [76]. However, there are many examples of marine organisms that achieve high adhesion strengths under water (e.g., barnacles and mussels) [90–92], so that submersion alone may not fully explain the low stress levels of cells.

A second key factor that reduces cell adhesion is long-range repulsion by the glycocalyx, a layer of glycoproteins and glycolipids on the outer cell surface [93, 94]. These surface molecules carry negative charges that inhibit adhesion between cells or negatively charged surfaces. The electrostatic repulsion by the glycocalyx is further enhanced by steric repulsion and osmotic effects [93]. Treatments reducing the negative charge on substrates were found to increase cell adhesion dramatically [95], and it is likely that excessive non-specific adhesion would lead to cell clustering and render cells unable to move. Cell locomotion is indeed enhanced on more negatively charged substrates [96]. Thus, while sufficient adhesion to diverse substrates via non-specific adhesion is essential for climbing animals to prevent detachment, the priority for cells may be exactly the opposite. Cells have to reduce or prevent non-specific adhesive interactions to maintain motility and to allow the controlled formation and release of specific adhesive contacts. Prevention of unwanted adhesion is also a common theme in the larger world of climbing animals. Many plant surfaces prevent insect adhesion with the help of epicuticular wax crystals or surface textures that trap lubricating water films [97]. In many insects, fields of microtrichia occur in regions immediately outside adhesive contact zones; it is likely that these surface structures are non-adhesive and facilitate detachment when the foot pad is rolled off, or in some systems prevent the self-matting of adhesive hairs (Fig. 3c) [98].

One consequence of the interplay of short-range attraction between cell adhesion molecules and long-range repulsion by the glycocalyx is that adhesion is concentrated locally in small adhesion domains such as focal contacts or desmosomes, which are connected intracellularly to filaments of the cytoskeleton [99]. This arrangement is functionally similar to adaptations in animal adhesive structures. The division of one large contact into many sub-contacts (contact splitting [100]) is one design principle of ‘hairy’ adhesive systems found in insects, spiders and geckos, which consist of dense arrays of microscopic hairs. Contact splitting can increase adhesion if the forces of individual contacts scale with their width or perimeter (comparing a smooth and a hairy toe pad of the same size, the hairy pad has a much smaller total contact area, but a much larger total contact perimeter), and if different contacts are loaded simultaneously so that a condition of ‘equal load sharing’ is achieved [101]. Cells are able to control the pulling forces on focal contacts by contractile stress fibers and remodeling of the actin fiber network [75, 102, 103]. Climbing animals can actively distribute loads between different legs or pads; but within each individual pad, the stress distribution depends mainly on the pad’s stiffness and mechanical design. The adhesion forces of many animals scale with contact area [88], suggesting that their pads are designed in a way that allows them to achieve uniform load distribution, but the underlying mechanisms are still unclear. Separate subcontacts also help for attachment on uneven substrates. Although gecko and insect adhesive hairs are made of relatively stiff keratin and cuticle, respectively, arrays of adhesive hairs are compliant and can maintain adhesion even on substrates with significant surface roughness [104, 105].

#### Adhesion and locomotion

One of the most apparent parallels between cell adhesion and whole-animal adhesion is that, in both systems, the contacts are *reversible* and *dynamic* to allow locomotion. When adhesive pads of climbing animals are sheared (pulled) towards the body, they adhere firmly, but they detach when pushed. A pull maximizes adhesion by increasing not only the adhesive contact area, but also the force per contact area [106].

Cells and animals are able to switch between different types of adhesive bonds, depending on whether weak temporary or strong permanent adhesion is required. Leucocytes provide a well-known example of an active change from weak/transient to stronger/more permanent adhesion [107]. Leucocytes moving from the circulatory system into infected tissues undergo states of weak adhesion, in which they 'roll' on the wall of venules, followed by firm adhesion to endothelial cells and transmigration through the endothelium, each involving different adhesion molecules (selectins and integrins) with varying binding affinities. The different steps of this cascade are triggered by the release of cytokines from infected tissues which trigger the expression of selectin molecules on the inner vessel wall, and by the release of chemokines which activate integrins on the leucocytes. While the shear force exerted by the blood flow is sufficient to maintain dissociation of selectins to induce cell rolling, it is too small to dislodge cells in the firm adhesion state [108]. There are some analogous cases of animals switching between different adhesive mechanisms for temporary and permanent adhesion. For example, limpets use a glycoprotein glue and achieve high adhesive strengths when stationary at low tide, but suction (achieving lower adhesive strengths) when moving around underwater at high tide [109]. However, most animals do not control adhesion by varying the chemistry of their adhesive bonds (this would probably be too slow for climbing animals), but by changing the geometry of the adhesive contact.

When climbing rapidly, geckos, spiders and insects are able to attach and detach their feet within tens of milliseconds. Although foot detachment can be very fast, it occurs without any measurable force peaks in geckos and insects [110]; [T. Endlein & W. Federle, unpubl. results]. The key principle underlying this impressive performance is the same across many different taxa and adhesive pad designs: climbing animals use shear forces (pulling and pushing) to turn adhesion on and off [111, 112]. A pull towards the body typically brings the pad into contact and maximizes adhesion, whereas a push leads to detachment. Adhesive setae of geckos, spiders and insects have oblique tips that require a pull to be bent slightly and come into full contact (Fig. 3c). If the setae are pushed or if the pulling force is released, all hairs of a pad return almost simultaneously to their non-adhesive default position, allowing effortless and rapid detachment [112, 113]. The smooth pads of ants and stick insects achieve very similar functionality via different mechanisms. When pulled, ant adhesive pads unfold and achieve a large contact area, but the pad recoils to a retracted default position when the pull is released (Fig. 3d) [114]. In stick insects, pads respond to pulls with a lateral expansion of the contact zone, likely driven by a hydrostatic mechanism in the outer cuticle [115]. An animal’s response to external forces is usually both passive and active. For example, the adhesive pads of ants can be unfolded both actively via a contraction of their claw flexor muscle or passively when a pulling force is acting on them. Passive ‘preflex’ reactions do not depend on the neuromuscular system and thus have the advantage that they can occur extremely rapidly, thereby preventing detachment by unexpected perturbations such as raindrops (Fig. 3f) [116].

Individual cells have an analogous ability to increase contact area in response to forces acting on them. Cells can react by changing the density of attractive and repellent cell adhesion molecules by exo- and endocytosis [117]. Moreover, pulling forces can reinforce focal adhesions [118] by stimulating them to grow along the axis of the externally applied force (Fig. 3g) [38]. Not only the adhesive contacts themselves can be adapted to external forces, but also their links to the cytoskeleton. Connections between focal adhesions and the cytoskeleton can be either cut by proteases or strengthened by new assembly [119]. It was found that the cytoskeleton itself increases its stiffness in response to forces acting on focal adhesions [120]. In contrast to the passive preflexes in pads of climbing animals, cells’ reactions are active and involve mechanotransduction. That is, cells sense mechanical stresses and convert them into intracellular signaling and biochemical reactions. While preflexes can double the adhesive contact area within less than a millisecond [121], the active nature of cells’ adhesion reaction implies that it is much slower and occurs within seconds or minutes [93, 122, 123].

In summary, the key principles of adhesion in cells and climbing animals are surprisingly similar, despite extreme differences in scale, structure and biological context. Both cells and climbing animals are able to resist detachment forces and distribute loads between different adhesive contacts. Although similar physical mechanisms are acting, adhesive stresses are much smaller in cells than in climbing animals, likely a consequence of the watery environment and the repulsion by the glycocalyx. The smaller stresses may help cells to maintain mobility despite their much larger surface-to-volume ratio. Dynamic and reversible adhesion is an essential requirement for both individual cells and climbing animals. The control of adhesion allows both to move and to react to varying environmental conditions. Adhesion control in climbing animals is faster than in cells, partly due to the passive (mechanical) reactions that operate in addition to active control. Future research on cells and climbing animals may uncover further similarities of their adhesive mechanisms.
